# Strain-Specific Anti-inflammatory Properties of Two *Akkermansia muciniphila* Strains on Chronic Colitis in Mice

**DOI:** 10.3389/fcimb.2019.00239

**Published:** 2019-07-05

**Authors:** Rui Zhai, Xinhe Xue, Liying Zhang, Xin Yang, Liping Zhao, Chenhong Zhang

**Affiliations:** ^1^State Key Laboratory of Microbial Metabolism, School of Life Sciences and Biotechnology, Shanghai Jiao Tong University, Shanghai, China; ^2^Department of Biochemistry and Microbiology, School of Environmental and Biological Sciences, Rutgers New Jersey Institute for Food, Nutrition, and Health, Rutgers University–New Brunswick, New Brunswick, NJ, United States

**Keywords:** *Akkermansia muciniphila*, strain-specificity, chronic colitis, DSS, Treg, gut microbiota

## Abstract

*Akkermansia muciniphila* is potential probiotic in that its type strain ATCC BAA-835 has beneficial effects upon obesity and diabetes. However, whether *A. muciniphila* can improve inflammatory bowel diseases (IBD), which is a form of chronic intestinal dysbiosis, is unknown. Hence, we used an isolated murine *A. muciniphila* strain (designated 139) and *A. muciniphila* type strain ATCC, to investigate their anti-inflammatory properties in cell models and in Dextran Sulfate Sodium (DSS)-induced chronic colitis of mice. *In vitro*, the two *A. muciniphila* strains exerted similar anti-inflammatory properties as they both reduced IL-8 production by TNF-α-stimulated HT-29 cells. However, neither of the strains showed capacity to increase the differentiation of regulatory T (Treg)-cells from CD4+ T cell populations significantly. *In vivo*, both *A. muciniphila* strains exerted anti-inflammatory effects on chronic colitis as they improved clinical parameters including spleen weight, colon inflammation index, and colon histological score. They also down-regulated the expression of the pro-inflammatory cytokines including TNF-α and IFN-γ in the colon of mice. However, the anti-inflammatory effects of strain ATCC were stronger than strain 139 in that ATCC significantly reduced spleen weight, colon inflammation index, and fecal lipocalin-2 content in mice with chronic colitis, while strain 139 was not. Dysbiosis of the gut microbiota was observed in mice with chronic colitis. Both *A. muciniphila* strains facilitated the normalization of the gut microbiota. The specific capacity of strain ATCC to modulate the differentiation of Tregs as well as increase production of short chain fatty acids, demonstrated strain-specific characteristics for these two *A. muciniphila* strains. This study suggests the potential beneficial effect of *A. muciniphila* on IBD and the importance of the future study of the function of *A. muciniphila* at the strain-level.

## Introduction

*Akkermansia muciniphila* is a commensal bacterium of the intestinal tract, which uses mucin as its sole carbon and nitrogen source. Under anaerobic conditions, the bacterium produces acetate and propionate within mucin (Derrien et al., 2004). The type strain of *A. muciniphila* (ATCC BAA-835) was isolated from the feces of a healthy human. This bacterium is a promising candidate for next-generation probiotics in that it has beneficial effects in animal models of human disease including obesity, diabetes, and alcoholic liver disease (Cani and de Vos, 2017; Plovier et al., 2017; Grander et al., 2018). And its membrane protein, Amuc_1100, strengthens gut permeability and improves metabolic disorders of mice (Plovier et al., 2017).

Despite obesity beneficial effects, controversies do exist regarding the effect of *A. muciniphila* on other diseases, including inflammatory bowel disease (IBD). IBD is a chronic inflammatory disorder related to the immune system and to the intestinal microbiota of the host (Maloy and Powrie, 2011). Many murine models of colitis induced by chemicals or by gene deficiency can be used to mimic the pathological or immunological features of IBD (Wirtz and Neurath, 2007). Several studies have found the abundance of *Akkermansia* to be decreased or unaltered in IBD patients (Png et al., 2010; Morgan et al., 2015; Lopez-Siles et al., 2018), while the abundance of *Akkermansia* demonstrated to be predominantly augmented in mice with colitis (Håkansson et al., 2015; Zhang et al., 2016). Extracellular vesicles of *A. muciniphila* ATCC BAA-835 have been shown to alleviate acute colitis in mice. However, the same strain was demonstrated to aggravate gut inflammation in gnotobiotic mice infected with *Salmonella typhimurium* (Ganesh et al., 2013; Kang et al., 2013). As such, the effect of *Akkermansia* on IBD remains unclear.

Most investigations of *A. muciniphila* have assessed only one strain, ATCC BAA-835. Genomic analysis of 39 *A. muciniphila* strains from the gut of mammals revealed notable genomic diversity, which implies functional specificity (Guo et al., 2017). Strain-specific physiological properties in both beneficial species and pathogens have been revealed in many studies (Myers et al., 2006; Wu et al., 2017; Pan et al., 2018). Thus, investigation of the physiological properties of various *A. muciniphila* strains may be of value.

Herein, the human-derived *A. muciniphila* strain ATCC BAA-835 and a novel murine *A. muciniphila* strain (designated as 139) were investigated for anti-inflammatory properties *in vitro* and *in vivo*. *In vitro*, both strains revealed anti-inflammatory characteristics but no immune-regulatory capacities. In the Dextran Sulfate Sodium (DSS)-induced chronic colitis murine model, ATCC BAA-835 significantly ameliorated intestinal inflammation, with increasing T regulatory cell (Treg) differentiation and short chain fatty acid (SCFA) production, while strain 139 did not show effect on the Treg differentiation and SCFAs production. However, both strains shifted the intestinal microbiota of mice. The work reported herein demonstrates the beneficial effects of *A. muciniphila* strains in chronic colitis as well as the existence of strain-specific anti-inflammatory properties of two *A. muciniphila* strains.

## Materials and Methods

### Isolation of *A. muciniphila* Strain

Fresh feces were collected from four specific pathogen free (SPF) 7-week-old C57/BL6 male mice (SLAC Inc., Shanghai, China) and suspended in anaerobic Ringer's solution (containing 0.05% L-cys). The suspensions were placed in an anaerobic work station (Don Whitley Scientific Ltd, Shipley, UK) within 10 min of harvest. Fecal matter was serially diluted 10-fold with Ringer's solution (containing 0.05% L-cys). The 10^−3^ dilution of suspension was placed onto mucin media plates (Derrien et al., 2004) and incubated under anaerobic condition (80% N_2_, 10% CO_2_, and 10% H_2_) at 37°C for 48 h until obvious colonies were observed. Isolated colonies (550) were purified by streaking three times on mucin media plates and identified as *A. muciniphila* by use of species-specific primers AM1 (5′-CAGCACGTGAAGGTGGGGAC-3′) and AM2 (5′-CCTTGCGGTTGGCTTCAGAT-3′) (Collado et al., 2007). Only three isolates were identified as *A. muciniphila*. Enterobacterial Repetitive Intergenic Consensus (ERIC) sequences of the genomes of the three isolates and *A. muciniphila* strain ATCC BAA-835 were amplified by polymerase chain reaction (PCR) using primers ERIC1 (5′-ATG TAAGCTCCTGGGGATTCAC-3′) and ERIC2 (5′-AAG TAAGTGACTGGGGTGAGCG-3′) (Vandamme et al., 1993). The three isolates shared the same ERIC-PCR profile and differed from strain ATCC BAA-835 (ATCC). One isolate (139) was randomly selected as representative and further identified by 16S rRNA gene sequencing, and identified as a new *A. muciniphila* strain.

### Culturing and Preparation of *A. muciniphila* Strains

*A. muciniphila* strain ATCC and 139 were cultured anaerobically (80% N_2_, 10% CO_2_, and 10% H_2_) in synthetic media to stationary phase (Plovier et al., 2017). For preparation of bacterial culture supernatant (BCS), bacterial density was set to 0.2 (OD 600) in unfermented synthetic media, then centrifuged at 8000 × g for 20 min. The BCS was collected in another tube, sterilized using a 0.22 μm filter (Merck Millipore, MA, USA) and stored at −80°C. Bacteria for gavage were collected after centrifugation, washed once with 5 ml of anaerobic phosphate buffered saline (PBS, pH = 7.4), then resuspended in anaerobic PBS to a density of 2 × 10^10^ cfu/ml. The suspension was distributed into 4 ml centrifuge tubes (150 μl/tube) to which was added 150 μl PBS with 50% glycerin, mixed thoroughly and stored at −80°C. Before gavage, the stored bacterial cells were defrosted anaerobically and diluted to 1 × 10^9^ cfu/ml by adding 2,700 μl of anaerobic PBS.

### Whole-Genome Sequencing and Analysis of *A. muciniphila* Strains

Whole-genome DNA of *A. muciniphila* strain 139 was extracted with the blood and cell culture DNA kit (Qiagen, USA) and sequenced with the PacBio RSll platform (Nextomics Biosciences, Wuhan, China). Subreads of the sequence were assembled into a single complete chromosome using the HGAP 2.3.0 pipeline (Chin et al., 2013). The genome sequence of *A. muciniphila* ATCC BAA-835 was downloaded from the NCBI nucleotide database with accession no. NC_010655.

The genome sequences of the two strains were adjusted to start with the replicational origin (location of *DnaA*) for comparison. Protein-coding sequences (CDSs), tRNAs, and rRNAs were predicted and annotated using the Prokka 1.12 pipeline (Seemann, 2014). Predicted CDSs were assigned into clusters of orthologous groups (COGs) using COG triangles (ftp://ftp.ncbi.nih.gov/pub/wolf/COGs/COGsoft/). The strain-specific CDSs of each strain were identified with mGenomeSubstractor (defined with H < 0.42) (Shao et al., 2010). Genomic alignment of the two *A. muciniphila* strains was visualized with strain-specific CDSs of each strain by BRIG (BLASTN with default parameters) (Alikhan et al., 2011).

### *In vitro* Anti-inflammatory Test

Human HT-29 cells were obtained from the Cell Bank of the Type Culture Collection of the Chinese Academy of Sciences (Shanghai, China) and cultured in Dulbecco's Modified Eagle's medium (DMEM) (Hyclone, CA, USA) with 10% fetal bovine serum (FBS) (Hyclone), 100 units/ml penicillin and streptomycin (Gibco, NY, USA) at 37°C in a humidified incubator with a 5% CO_2_/air atmosphere. 10% (v/v) BCS of each strain was proved to not effect the viability of HT-29 cells by 3-(4,5-dimethyl-2-thiazolyl)-2,5-diphenyl-2-H-tetrazolium bromide (MTT) assay. Cells were seeded into 24-well tissue culture plates at a density of 1 × 10^5^ cells/well in 1 ml DMEM. The medium was changed every day. After culturing for 7 days, cell culture medium was changed to FBS-free culture medium and cells cultured for an additional 24 h. Monolayer-confluent HT-29 cells were rinsed with PBS (Hyclone) once and then simulated with human recombinant 10 ng/ml TNF-α (PeproTech, USA) in DMEM added 10% (v/v) BCS, or 10% unfermented synthetic medium, or 10% DMEM for 8 hours. Cells without TNF-α served as controls. After incubation, culture medium was collected and centrifuged at 4000 × g for 5 min. Supernatants were collected for measurement of IL-8 using Human IL-8/CXCL8 Quantikine ELISA kits (R&D systems, MN, USA).

### *In vitro* Immune-Regulatory Test

Mesenteric lymph nodes (MLNs) were obtained from healthy SPF 10-week-old C57/BL6 male mice (SLAC Inc., Shanghai, China), then washed once and collected in PBS (Hyclone). CD4+ cells were separated from total cells using the Dynal® Mouse CD4 Cell Negative Isolation kit (Invitrogen, CA, USA). The CD4+ cells were placed in 96-cell tissue culture plates at a density of 1 × 10^6^ cells/well. CD4+ cells were co-cultured with 10% (v/v) BCS, or 10% unfermented synthetic medium, or 10% PBS (control) in DMEM with 5 ng/ml human TGF-β (R&D systems) and 10 ng/ml recombinant mouse IL-2 (R&D systems) for 72 h. The medium was changed every 36 h. Cells were collected by centrifugation at 1000 × g for 3 min (4°C), washed once, and resuspended in magnetic-activated cell sorting (MACS) buffer (PBS + 0.5 % BSA + 2 mM EDTA, pH = 7.2). The number of Foxp3+ Treg cells was assessed as described in Flow Cytometry.

### Flow Cytometry

Cells were blocked with anti-CD16/CD32 antibody (Biolegend, CA, USA), then surface stained with anti-CD3 antibody (FITC) (Biolegend) and anti-CD4 antibody (PE) (Biolegend) in MACS buffer for 45 min. After surface staining, cells were washed twice with PBS, stained with Fixable Viability Dye eFluor® 780 (FVD) (ebiosciences, CA, USA) in PBS for 30 min and washed twice with MACS buffer. Next, cells were fixed and permeabilized using the Mouse Foxp3 Buffer Set (ebiosciences) for 2 h, then stained with anti-Foxp3 antibody (APC) (Biolegend) for 45 min. Stained cells were washed twice with MACS buffer, then resuspended in MACS buffer, and assessed by the Beckman CytoFLEX flow cytometer (Beckman, CA, USA) with analysis by FlowJo vX.0.7 software (FlowJo LLC, OR, USA).

### Short-Chain Fatty Acids (SCFAs) Profiling

For measurement of SCFAs in BCS, 200 μl of BCS was acidified by adding 0.1 ml of 50% (v/v) sulfuric acid. After vortexing and standing for 2 min, the organic acids were extracted by adding 0.4 ml of diethyl ether. Then the concentration of SCFAs was measured using the Agilent 6890 (Agilent Technologies, CA, USA) with flame ionization, thermal conductivity detectors, and capillary column. For the determination of SCFAs in cecum content, 200 mg cecum content was homogenized with 1 ml PBS and centrifuged at 16000 × g for 15 min (4°C). The supernatants were filtered through 0.22 μm filters. Determination of SCFAs in BCS was as described above.

### Animal Trial

All SPF C57/BL6 male mice were purchased from SLAC Inc. (Shanghai, China). All the animal experimental procedures were approved by the Institutional Animal Care and Use Committee of the School of Life Sciences and Biotechnology, Shanghai Jiao Tong University (No. A2018003). Preliminary experiment was performed with 12 SPF C57/BL6 mice (male, 8-week-old) to ensure that gavaged *A. muciniphila* strains passed viably through the murine gut, the content change of *A. muciniphila* in feces was assessed with *A. muciniphila* species-specific primers mentioned before. For the formal experiment, 40 SPF C57/BL6 mice (male, 8-week-old) were randomly assigned to four groups (10 mice/group, 2 mice/cage). As shown in Supplementary Figure S5, three-rounds of treatment with 3% DSS were used to induce chronic colitis, with each round of treatment for 3 days. The mice were gavaged with 200 μl (2 × 10^8^ cfu) bacterial cells suspended in PBS with 2.5% glycerin (DSS+ATCC and DSS+139 groups), or PBS with 2.5% glycerin (NC group and DSS group) per day from the beginning to the end of the experiment. Body weight of mice was measured at day 0, 3, 9, 14, 17, 23, 28, 31, 36, 41, 46, and 56. Fecal samples were collected at day 31, 36, 41, 46, and 56, then stored at −80°C. Fecal lipocalin-2 content was measured with a Human Lipocalin-2/NGAL Quantikine ELISA kit (R&D Systems). Half of the mice of each group were sacrificed randomly on day 46 and day 56. A 10-day window was set to resolve colitis. After being sacrificed, tissues were weighed and collected, stored at −80°C. MLNs of mice were collected and stored in PBS (4°C), and Foxp3+ Treg cells measured as described in Flow Cytometry.

For the colons of mice, the distal portion was fixed in 4% paraformaldehyde, sectioned, and stained with hematoxylin-eosin (HE). Histological scoring was performed as previously described (Welz et al., 2011). The RNA of the middle portion of the colon was extracted and used to quantify the expression of specific genes.

### RNA Extraction and Real-Time Quantitative PCR (qRT-PCR)

The total RNA of colons was extracted using the RNeasy Mini Kit (Qiagen, Duesseldorf, Germany). The DNA was removed using DNase I (Invitrogen), then the RNA was reverse-transcribed to cDNA using the SuperScriptTM system (Invitrogen). qRT-PCR was performed with the LightCycler96 (Roche, Geneva, Switzerland) using iQ SYBR Green Supermix (BIO-RAD, CA, USA). The primer sequences are listed in Supplementary Table S1.

### Fecal 16S rRNA Gene V3-V4 Region Sequencing

DNA extraction of all fecal samples was performed as previously described (Godon et al., 1997). The construction of the sequencing library of the V3-V4 region of the 16S rRNA gene, sequencing with the Illumina MiSeq platform (Illumina, Inc., CA, USA), and the quality control of raw data were as previously described (Zhang et al., 2016). The high-quality sequences were classified into their corresponding Operational Taxonomic Units (OTUs) using the Usearch global alignment algorithm at a 97% cutoff for identity (Edgar, 2010). Representative sequences for all OTUs were assigned to taxonomy using q2-feature-classifier in QIIME 2 (Bokulich et al., 2018). The sequences of each sample were subsampled to 12,000 (1,000 permutations) to equalize differences in sequencing depth among samples. Richness and diversity of microbiota in each sample were assessed with observed OTUs and Shannon diversity index calculated in QIIME 1.8, respectively, and the statistical differences were analyzed with one-way ANOVA test (Caporaso et al., 2010). Principal coordinate analysis (PCoA) was performed using the rarefaction OTU table. The statistical significance among the gut microbiota composition of different groups was analyzed with multivariate analysis of variance (MANOVA) with MATLAB R2017a (The MathWorks Inc., MA, USA). Both PCoA and MANOVA clustering analyses were calculated with Bray-Curtis distance based on OTU abundance. A sPLS-DA model (Lê Cao et al., 2011) was established to identify the OTUs contributing to the separation of gut microbiota of the NC group and the DSS group at day 31 using “mixomics” R package (Rohart et al., 2017) in R version 3.5.1 (www.r-project.org). The heatmap of 39 OTUs identified with a sPLS-DA model was generated in R version 3.5.1. The heatmap shows the abundance variation of 35 OTUs in four groups and five time points generated in MATLAB R2017a.

### Statistical Analysis

Statistical analysis was carried out with GraphPad Prism version 7.0a (GraphPad Software, Inc., CA, USA). One-way ANOVA test was used to determine the statistical significance of the physiological data. Differences were considered significant when *P* < 0.05. Mann-Whitney *U* test (two-tailed) was used to analyze variations in the relative abundance of specific OTU between the NC and the DSS group, or the NC and the DSS+ATCC group, or the NC and the DSS+139 group. Then the stack of *P*-values was adjusted by false discovery rate estimation using the two-stage linear step-up procedure of Benjamini, Krieger, and Yekutieli to determine the adjusted *P*-value (Benjamini et al., 2006). Differences were considered statistically significant when *P* < 0.05.

### Sequence Data Accession Numbers

The 16S rRNA gene sequence of *A. muciniphila* strain 139 has been submitted to the Genebank with the accession no. MK578203. The whole-genome sequence of *A. muciniphila* strain 139 has been submitted to the NCBI under accession no. CP036293.The raw Illumine sequence data generated in this study are available in the sequence read archive (SRA) at NCBI under accession no. SRP187320. All the data were released in public.

## Results

### Genomic Characterization of *A. muciniphila* Strains ATCC and Murine Strain 139

**Figure 1:**
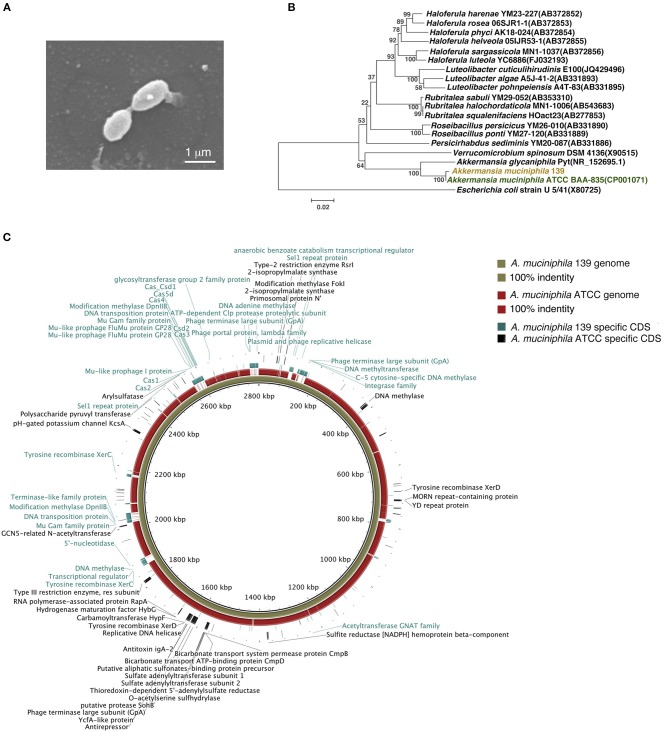
Identification of murine *A. muciniphila* strain 139 and its genomic comparison with *A. muciniphila* strain ATCC. **(A)** Electron micrograph of strain 139. **(B)** Phylogenetic relationships of the strain 139 with its relatives based on 16S rRNA gene sequences, and *Escherichia coli* U5/41 was used as an outgroup. The tree was constructed using the Neighbor-Joining method in MEGA6. The bar indicates sequence divergence. **(C)** Genomic comparison of the chromosomes of *A. muciniphila* strain ATCC and 139. Both sequences are started from the predicted replication origin. From inner to outer: (1) contig of strain 139, (2) the identity of genome of strain ATCC blast on the strain 139 based on BLASTN, and (3) the strain-specific CDSs of strain 139 and strain-specific CDSs of strain ATCC. The function of the annotated strain-specific CDSs are also labeled.

To investigate the anti-inflammatory characteristics of a different *A. muciniphila* strain, a newly isolated murine *A. muciniphila* strain (designated 139) was assessed. It was cultured to a stationary phase of growth for 40 h in liquid synthetic medium (Supplementary Figure S1). Similar to strain ATCC, strain 139 was oval-shaped with no flagella or other appendages as judged by scanning electron microscopy (Derrien et al., 2004). Its size was approximately (0.9–1.0) × (0.4–0.5) μm (Figure 1A). The nearest neighbor of strain 139, based on 16S rRNA, was *A. muciniphila* strain ATCC (similarity 99.79%, Figure 1B).

The genomes of strain ATCC and 139 shared high homology by nucleotide sequence (91% similarity) and G+C content (55.80% for strain ATCC and 55.74% for strain 139) (Supplementary Figure S2A). A comparison of general genomic features is shown in Supplementary Table S2. For COGs, the two *A. muciniphila* strains shared the most functional category genes (including *Amuc_1100*), which confirms the high genomic similarity of the two strains (Supplementary Figure S2B). However, strain 139 exhibited more functional category genes in 15 COG classifications while strain ATCC exhibited more functional category genes in four COG classifications, which implies distinct genomic differences.

To compare the genomes of the two strains at nucleotide level and to identify their strain-specific CDSs, we aligned the genomes (Figure 1C). Except for large-scale conserved sequences, several “gaps” were identified with <50% homology. Specifically, 278 and 125 CDSs were identified to be unique to the genomes of strain 139 and strain ATCC, respectively. Therefore, in spite of the high degree of similarity, the genomes of these two *A. muciniphila* strains contained strain-specific genes that may contribute to differences in physiological function.

### *A. muciniphila* Strains Demonstrated Similar Anti-inflammatory *in vitro*

**Figure 2:**
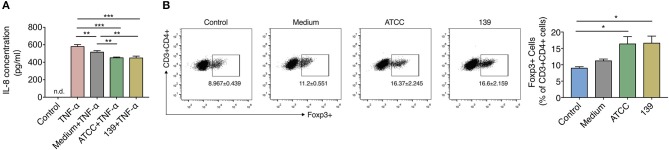
BCS from *A. muciniphila* ATCC and 139 exert anti-inflammatory but no immune-modulatory properties *in vitro*. **(A)** IL-8 concentration produced by HT-29 cells. Control, negative control without TNF-α stimulation; n.d., non-detectable. **(B)** Foxp3+ Tregs in the CD4+ T cells (CD3+ CD4+) detected by flow cytometry. Cytokines hTGF-β and rmIL-2 were added to facilitate the expression of Foxp3+ Treg cells. Data are the frequency of the Foxp3+ Treg cells among the CD3+ CD4+ cell population. Control, cells without cytokines. All the data are shown are means ± s.e.m., *n* = 3 for all groups. Values of each group were calculated by one-way analysis of variance (ANOVA) followed by Tukey's *post-hoc* test. *P < 0.05, **P < 0.01, ***P < 0.001.

To investigate the anti-inflammatory properties of the two *A. muciniphila* strains *in vitro*, we co-cultured TNF-α-stimulated human HT-29 cells with 10% (v/v) BSC of the two strains. IL-8, which can be produced by epithelial cells and is a key mediator associated with inflammation (Harada et al., 1994), was used to reflect the degree of inflammation in many *in vitro* studies including this (Li et al., 2019). After co-culture with the BSC of strain ATCC or 139, TNF-α stimulated HT-29 cells produced much less IL-8 than cells co-cultured with unfermented synthetic media (Figure 2A), which implies that both strains produce some substance(s) that inhibited IL-8 secretion of inflamed HT-29 cells.

CD4+ cells were sorted from MLNs of mice to investigate whether the two *A. muciniphila* strains could regulate the differentiation of immune cells. Result showed the differentiation of Foxp3+ Tregs prominently increased in the CD4+ T cells (CD3+CD4+) co-cultured with 10% BCS of strain ATCC and strain 139 when compared to cells co-cultured with PBS (control) (Figure 2B). However, when compared to the medium, neither strain ATCC nor strain 139 significantly augmented the differentiation of Foxp3+ Treg cells (Figure 2B). Thus, both the strains did not show strong immune-regulatory properties *in vitro*.

Since SCFAs are closely related to the anti-inflammatory and immune response, the content of SCFAs in BCSs of the two *A. muciniphila* strains was assessed. After fermentation in synthetic medium to stationary phase, both strains produced acetate and propionate by utilizing substances within the synthetic medium. However, the production of other kinds of SCFAs like butyrate were not found (Supplementary Figure S3).

Based on these experiments, *A. muciniphila* strain ATCC and 139 shared capacities to reduce the inflammation and produce acetate and propionate *in vitro*, while neither of them showed capacity to facilitate the differentiation of Treg cells *in vitro*.

### *A. muciniphila* Strains Ameliorate Murine Chronic Colitis in a Strain-Specific Manner

As the two strains demonstrated similar anti-inflammatory and immune-regulatory properties *in vitro*, a murine model was used to investigate the effects of these two *A. muciniphila* strains in chronic colitis. Preliminary experiments showed the relative abundance of the two *A. muciniphila* strains to maximally increase 4 h after gavage and with a gradual decrease to original levels by 24 h after gavage (Supplementary Figure S4). The experimental design is shown in Supplementary Figure S5. Mice in the NC group drank only water. Mice in the DSS, DSS+ATCC, and DSS+139 groups had chronic colitis induced by drinking water with 3% DSS for three cycles, each cycle 3 days in duration. Mice in the NC and DSS groups were gavaged with PBS containing 2.5% glycerin, every day. Mice in the DSS+ATCC and DSS+139 groups were gavaged every day with strain ATCC and 139, respectively.

**Figure 3:**
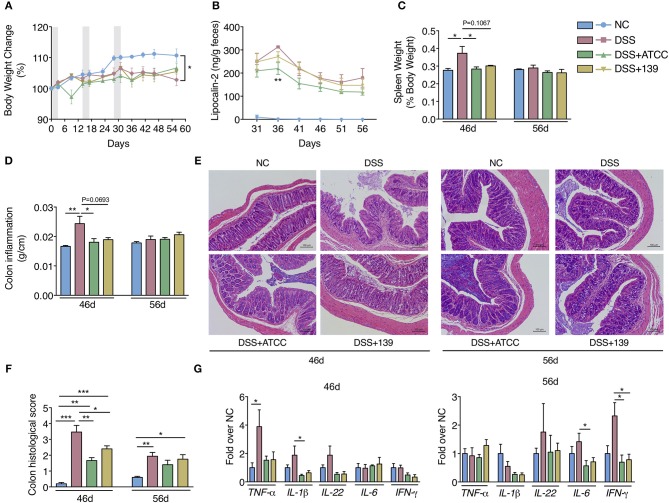
*A. muciniphila* strain ATCC and 139 showed strain-specific anti-inflammatory effects on the chronic colitis of mice. **(A)** Body weight change of mice (normalized to the original weight). Periods of DSS treatment were shadowed. **(B)** Fecal lipocalin-2 contents. **(C)** Spleen weight (ratio to body weight). **(D)** Conlon inflammation index (colon weight/length). **(E)** Representative hematoxylin-eosin stained sections of distal colon at day 46 and 56 (200×). **(F)** Histological scores of colon sections. **(G)** Colon mRNA expression of several pro-inflammatory mediators relative to β-actin in mice. For body weight and fecal lipocalin-2 level, *n* = 10 per group before 46d time point, *n* = 5 per group from 46d time point. For other data, *n* = 5 per group for each time point. Data are shown as means ± s.e.m. Values of each group were calculated by one-way analysis of variance (ANOVA) followed by Tukey's *post-hoc* test. For graph **(A,C–G)** *P < 0.05, **P < 0.01, ***P < 0.001. For graph **(B)**, **P < 0.01 compared with DSS group.

By day 31, mice in the DSS group began to gain less weight with higher fecal lipocalin-2 content than mice in the NC group (Figures 3A,B). Spleen weight and the colon inflammation index (colon weight/colon length) of mice in the DSS group were significantly higher than mice in the NC group at day 46 (Figures 3C,D). More severe mucosal damage and inflammatory infiltration with higher colon histological scores were observed in mice in the DSS group compared to the NC group at both days 46 and 56 (Figures 3E,F). Significant up-regulation of typical pro-inflammatory mediators TNF-a, lL-1β, IFN-γ, and IL-6 were detected in the colon of mice in the DSS group (Figure 3G). Compared with the mice in the DSS group, inflammation in the DSS+ATCC and DSS+139 groups resolved more quickly. These mice exhibited reduced spleen weight, colon inflammation index, and colon histological score at day 46 (Figures 3C,D,F). At day 56 the mice gained more weight with down-regulation of typical pro-inflammatory mediators (Figures 3A,G). However, the reduction of spleen weight and colon inflammation index were only significant for the DSS+ATCC group when compared to mice in the DSS group (Figures 3C,D). The fecal lipocalin-2 content of mice in the DSS+ATCC group was significantly lower than mice in the DSS group at day 36 and only the histological score of mice in the DSS+ATCC group recovered to the level of the NC group by the end of the experiment (Figures 3B,F). Thus, both *A. muciniphila* strains accelerated the recovery of chronic colitis in mice, with results only significant for strain ATCC.

**Figure 4:**
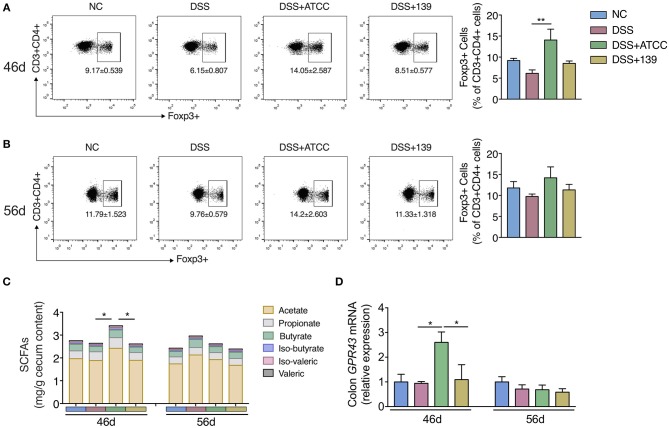
*A. muciniphila* strain ATCC promoted the MLNs Treg cells differentiation and SCFAs production of mice. **(A,B)** The frequency of Foxp3+ Treg cells among the CD4+ T cells (CD3+ CD4+ cells) in MLNs of mice at day 46 (*n* = 5 per group) and day 56 (*n* = 4 per group) detected by flow cytometry. **(C)** The content of SCFAs (acetate, propionate, butyrate, iso-butyrate, iso-valeric, and valeric) in cecum content of mice at day 46 and day 56 (*n* = 5 per group). **(D)** mRNA expression of *GPR43* in colon of mice (*n* = 5 per group). All the data are shown as mean ± s.e.m. Values of each group were calculated by one-way analysis of variance (ANOVA) followed by Tukey's *post-hoc* test. *P < 0.05, **P < 0.01.

Then we explored whether the two strains could impact the differentiation of Foxp3+ Treg cells in MLNs of mice. Only strain ATCC increased the conversion of CD4+ T cells (CD3+ CD4+) to Foxp3+ Treg in MLNs of mice at day 46 (Figure 4A). While no significant difference in Treg differentiation was observed at day 56 (Figure 4B). Further, a significant increase in total SCFAs (acetate, propionate, butyrate, iso-butyrate, iso-valeric, and valeric) was observed in the cecum with an up-regulation of *GPR43* in the colon of mice gavaged with strain ATCC (Figures 4C,D). However, neither of the strains showed strong capacity to strengthen the intestinal barrier as they did not up-regulate the expression of genes associated with the intestinal barrier function (Supplementary Figure S6). Therefore, strain ATCC had a greater anti-inflammatory capacity that included an increase in the differentiation of Tregs and the production of SCFAs in the gut of mice with chronic colitis.

### Both *A. muciniphila* Strains Promoted Normalization of the Gut Microbiota

**Figure 5:**
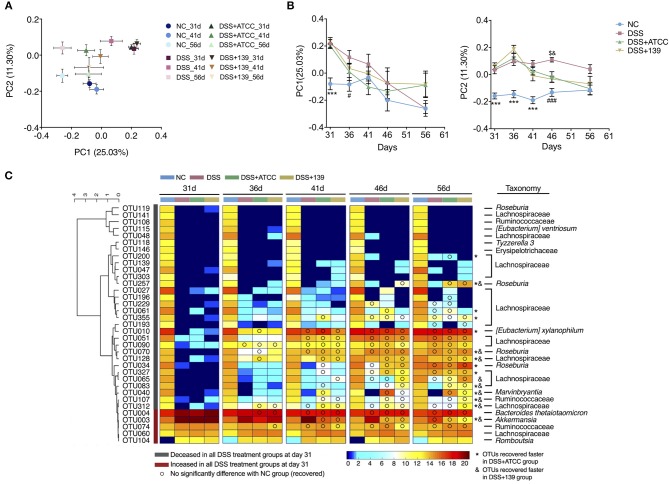
Modulation of gut microbiota by DSS treatment or by DSS treatment and the two strains of *A. muciniphila*. **(A)** Bray-Curtis PCoA plot of day 31, 41, and 56 based on OTU abundance. **(B)** Variation of gut microbiota structure on PC1 and PC2 based on Bray-Curtis distance, *n* = 10 for each group before the 46 day time point, *n* = 5 for each group. Data are shown as means ± s.e.m. Values of each group were calculated by one-way analysis of variance (ANOVA) followed by Tukey's *post-hoc* test. ***P < 0.001 compared to all three groups treated with DSS, ^*###*^*P* < 0.001 compared to the DSS group, ^*$*^*P* < 0.05 compared to the DSS+ATCC group, ^&^*P* < 0.05 compared to the DSS+139 group. **(C)** Heat map of 35 OTUs after DSS treatment during the alleviation period of mouse colitis. The color of the spots in the panel represent the mean relative abundance (log-transformed) of the OTUs in each group. The OTUs are clustered according to the Spearman correlation coefficient, *n* = 10 for each group before the 46 day time point, *n* = 5 for each group. The relative abundance of each DSS-treated group was compared to the NC group by Mann-Whitney *U* test. Compared to the relative abundance of the NC group at day 31, the taxonomy of OTUs increased significantly in all DSS-treated groups colored red, decreased significantly in all DSS-treated groups colored gray. OTUs recovered faster in mice gavaged with strain ATCC and are marked with *. OTUs recovered faster in mice gavaged with strain ATCC and are marked with &.

To determine if the gut microbiota of mice was affected by DSS treatment or the two *A. muciniphila* strains, 16S rRNA V3-V4 region sequencing of fecal microbes (at day 31, 36, 41, 46, and 56) was performed. At day 31, the Shannon index (diversity) and observed OTUs (richness) of the gut microbiota of all mice treated with DSS significantly decreased in comparison to mice of the NC group (Supplementary Figure S7A). DSS treatment predominantly shifted the gut microbiota of mice from day 31 (*P* < 0.05, MANOVA test compared with the NC group) (Supplementary Figures S7C,D). In addition, the gut microbiota of mice in the DSS+ATCC and the DSS+139 groups were significantly different in comparison to the DSS group from day 41 (*P* < 0.05, MANOVA test compared to the DSS group) (Figure 5A; Supplementary Figures S7E–G). Thus, the gut microbiota was changed by both DSS treatment and the two *A. muciniphila* strains.

With gradual recovery from colitis, the decreased Shannon index of mice in the DSS+ATCC and the DSS+139 groups increased to the level of the NC group at day 36, which was quicker than the DSS group. The decreased OTUs of mice in the DSS+ATCC group recovered to the same level as the NC group at day 41, which was quicker than the DSS and the DSS+139 groups (Supplementary Figure S7A). With regard to the structure of the gut microbiota and based on the Bray-Curtis PCoA plot, mice in the DSS, DSS+ATCC, and DSS+139 groups tended toward the NC group along the PC1 and PC2 axes after day 31 (Figure 5A; Supplementary Figure S7B). However, the gut microbiota composition of mice in the DSS+ATCC and DSS+139 groups was not significantly different from the NC group along PC1 axis from day 36 and along PC2 axis from day 46, which was earlier than the DSS group (Figure 5B). Hence, both the *A. muciniphila* strains accelerated the normalization of the diversity and structure of the gut microbiota, with strain ATCC accelerating the richness recovery of the murine gut microbiota.

To detail changes in gut microbiota, 39 OTUs were identified that contributed to the distinction of gut microbiota structure in the DSS group compared to the NC group by the sPLS-DA model (validated by the leave-one-out method, Supplementary Figure S8). Among these 39 OTUs, the relative abundance of 35 OTUs was significantly changed in mice treated with DSS. Thirty of these 35 OTUs (27 OTUs of the Lachnospiraceae family) decreased significantly in the three groups treated with DSS and five of these including OTU3 (100% similarity to *A. muciniphila* based on 16S rRNA v3-v4 sequence) increased significantly in the three groups treated with DSS at day 31 (Figure 5C). With the recovery from chronic colitis, the relative abundance of some OTUs recovered as well (FDR adjusted *P* ≥ 0.05). To the end of the experiment (day 56), 15 OTUs recovered in the DSS group, 21 OTUs recovered in the DSS+ATCC group, and 18 OTUs recovered in the DSS+139 group (Figure 5C). OTU200 (Lachnospiraceae) and OTU27 (Lachnospiraceae) recovered only in mice gavaged with strain ATCC (Figure 5C). Comparing the time when OTUs recovered, 8 OTUs including; OTU257 (*Roseburia*), OTU70 (*Roseburia*), OTU128 (Lachnospiraceae), OTU40 (*Marvinbryantia*), OTU107 (Ruminococcaceae), OTU312 (Lachnospiraceae), and OTU3 recovered faster in both the DSS+ATCC and the DSS+139 groups. Seven OTUs including; OTU200 (Lachnospiraceae), OTU27 (Lachnospiraceae), OTU355 (Lachnospiraceae), OTU61 (Lachnospiraceae), OTU10 (*[Eubacterium] xylanophilum*), OTU34 (*Roseburia*), and OTU327 (Lachnospiraceae) recovered faster only in the DSS+ATCC group. OTU65 (Lachnospiraceae) recovered faster only in the DSS+139 group. Therefore, both strains of *A. muciniphila* facilitated the normalization of the gut microbiota composition of mice shifted by DSS treatment. However, more OTUs recovered in mice gavaged with strain ATCC than strain 139.

## Discussion

The gut microbiota has become a key entry point in the study of the pathogenesis and the therapeutic treatment of IBD. Many microbes of the gut have been identified to contribute to the formation or the recovery from IBD (e.g., *Bacteroides*) based on their performance in animal models of colitis (Bloom et al., 2011; Ni et al., 2017). Herein, we demonstrated two *A. muciniphila* strains, strain ATCC and strain 139 showed anti-inflammatory properties *in vitro*, ameliorated chronic murine colitis in a strain-specific manner.

Cross-talk between microbe-derived metabolites and the immune system is essential to maintain host intestinal homeostasis (Gonçalves et al., 2018). Previously, *Bifidobacterium animalis* and *Clostridium butyricum* were shown to attenuate intestinal inflammation by producing typical SCFAs (acetate, propionate, and butyrate) by promoting the differentiation of Tregs (Hayashi et al., 2013; Hidalgo-Cantabrana et al., 2016). For the *A. muciniphila* strain ATCC, both the previous and current study demonstrated production of acetate and propionate utilizing typical substrates (Derrien et al., 2004). In addition, gavaging *A. muciniphila* strain ATCC enabled the mice to regain faster intestinal SCFA producers, e.g., family Lachnospiraceae species *Roseburia* and *Marvinbryantia* (Rey et al., 2010; Meehan and Beiko, 2014; Tamanai-Shacoori et al., 2017). Each could increase the production of SCFAs in the gut of mice with chronic colitis as observed in this study. Moreover, SCFAs can activate G-protein coupled receptor *GPR43*, which plays a crucial role in the regulation of immune responses including an increase in the number of colonic Foxp3+ Tregs and an inhibition of the pro-inflammatory response of mice with colitis (Maslowski et al., 2009; Kim et al., 2013; Smith et al., 2013). We also observed up-regulated colonic *GPR43*, increased differentiation of Foxp3+ Tregs, and down-regulated expression of typical pro-inflammatory mediators of mice. Thus, we demonstrated *A. muciniphila* strain ATCC to ameliorate chronic colitis of mice through the cross-talk of microbe-derived SCFAs and Foxp3+ Treg cells.

The cross-talk among bacterial metabolites (e.g., SCFAs), the immune system, and host intestinal homeostasis are closely related to the gut microbiota. Dysbiosis of the gut microbiota decreases some typical bacterial taxa like *Roseburia* and increases some typical bacterial taxa like *Bacteroides fragilis*, which is often observed in IBD patients and mouse models (Takaishi et al., 2008; Machiels et al., 2014; Zhou and Zhi, 2016). Previously, *Lactobacillus* was shown to modulate the gut microbiota, producing an anti-inflammatory effect in mice with colitis (Rodríguez-Nogales et al., 2017). In the current work, we also found two *A. muciniphila* strains to facilitate the normalization of specific intestinal microbes that were disordered in mice with chronic colitis (e.g., *Roseburia* and *Marvinbryantia)*. Thus, we assessed the ability of the two *A. muciniphila* strains to attenuate chronic colitis and to modulate the dysbiosis of the gut microbiota. However, the causal relationship needs further investigation.

Many studies, including the current one, have confirmed the contribution of specific gut microbes to intestinal homeostasis, with the identification of specific bacterial strains. Previously, different strains of *Bifidobacterium longum* were shown to inhibit acute colitis of mice in a strain-specific manner (Srutkova et al., 2015). In recent work, two *Lactobacillus murinus* strains showed differing anti-inflammatory properties in a Caco-2 cell model (Pan et al., 2018). Herein, although we found the similar anti-inflammatory of the two *A. muciniphila* strains *in vitro*, we also identified strain-specific genomic characteristics as well as differing anti-inflammatory properties in mice with chronic colitis. Seregin et al. (2017) demonstrated another murine *A. muciniphila* strain to exacerbate colitis in IL10−/− mice. Those observations, with those reported herein, demonstrate strain-specific physiological functions for different *A. muciniphila* strains.

We showed *A. muciniphila* ATCC to ameliorate the chronic colitis of male mice, although it has been reported to have no effect on DSS-induced acute colitis of female mice (Kang et al., 2013). Previously, mouse gender has been shown to influence the pathogenicity of DSS and the therapeutic effect of resveratrol on DSS-induced colitis in mice (Wagnerova et al., 2017). It is important to note that the pathological features of chronic colitis and acute colitis are not exactly the same (Perše and Cerar, 2012; Wirtz et al., 2017). Thus, the gender and phase of DSS-induced colitis in mice may influence the effects of the *A. muciniphila* strain ATCC. However, this speculation requires further verification. In this study, fewer *A. muciniphila* were found in mice gavaged with *A. muciniphila* strains. We have confirmed that the gavaged strains did reach the gut of the mice and remained for hours. There are three possible explanations. First, the change in the relative abundance of *A. muciniphila* was influenced by the change in the gut environment but did not directly influence the gavaged strains as the mucus of mice is broken after the development of colitis, which releases more liberated mucus glycans than can be utilized by *A. muciniphila* (Glymenaki et al., 2017). Second is strain-specific properties of *A. muciniphila*. Third is strain competition among *A. muciniphila* similar to non-toxigenic *Bacteroides fragilis* that can limit the colonization of pathogenic enterotoxigenic *Bacteroides fragilis* in the gut of mice (Hecht et al., 2016). These three explanations will be investigated in further studies.

In conclusion, we have shown strain-specific properties for two *A. muciniphila* strains that attenuate chronic intestinal inflammation and normalize the gut microbiota of mice. Our study not only suggests *A. muciniphila* strains, in particular strain ATCC, to be potential probiotics for human IBD, but also highlights the importance of the study of the relationships between the gut microbiota and host health at the bacterial strain-level.

## Data Availability

Publicly available datasets were analyzed in this study. These data can be found here: https://www.ncbi.nlm.nih.gov/nuccore/CP036293; https://www.ncbi.nlm.nih.gov/nuccore/MK578203.1/; https://www.ncbi.nlm.nih.gov/sra/SRP187320.

## Ethics Statement

This study was carried out in accordance with the recommendations of Guidelines on the treatment of laboratory animals, Ministry of Science and Technology of the People's Republic of China. The protocols was approved by the Institutional Animal Care and Use Committee of the School of Life Sciences and Biotechnology, Shanghai Jiao Tong University with No. A2018003.

## Author Contributions

CZ conceived and designed the study. XX isolated *A. muciniphila* strain 139. RZ and LYZ conducted the animal trial and sample collection. RZ conducted the cell models experiment and the RNA isolation and physiological data analysis. RZ and XY prepared the DNA and conducted the sequencing of 16S rRNA gene. RZ conducted the sequencing data analysis. RZ, CZ, and LPZ wrote and revised the manuscript.

### Conflict of Interest Statement

The authors declare that the research was conducted in the absence of any commercial or financial relationships that could be construed as a potential conflict of interest.
