# Supplementary material for: Strain-Specific Anti-inflammatory Properties of Two Akkermansia muciniphila Strains on Chronic Colitis in Mice
Source: Front Cell Infect Microbiol. 2019 Jul 5;9:239. doi: 10.3389/fcimb.2019.00239 (PMC6624636; doi:10.3389/fcimb.2019.00239)
Supplement: Supplementary file 1 [file Data_Sheet_1.pdf]

frontiers

Supplementary Materials

Supplementary tables and Figures

Supplementary Table S1. Sequences of primers used in research

Name	Sequences
IL-6	F: 5' -TAGTCCTTCCTACCCCAATTTCC- 3'<br>R: 5' -TTGGTCCTTAGCCACTCCTTC- 3'
IL-1β	F: 5' -TCCATGAGCTTTGTACAAGGA- 3'<br>R: 5' -AGCCCATACTTTAGGAAGACA- 3'
TNF-α	F: 5' -GTCCCCAAAGGGATGAGAAGTT- 3'<br>R: 5' -GTCCCCAAAGGGATGAGAAGTT- 3'
IFN-γ	F: 5' -GCCACGGCACAGTCATTGA- 3'<br>R: 5' -TGCTGATGGCCTGATTGTCTT- 3'
IL-22	F: 5' -GTCAACCGCACCTTTATGCT- 3'<br>R: 5' -GAACAGTTTCTCCCCGATGA- 3'
GPR43	F: 5' -CTTGATCCTCACGGCCTACAT- 3'<br>R: 5' -CCAGGGTCAGATTAAGCAGGAG- 3'
MUC2	F: 5' -GCTGACGAGTGGTTGGTGAATG- 3'<br>R: 5' -GATGAGGTGGCAGACAGGAGAC- 3'
Occludin	F: 5' -ATGTCCGGCCGATGCTCTC- 3'<br>R: 5' -TTTGGCTGCTCTTGGGTCTGTAT- 3'
Cldn3	F: 5' -TCATCGGCAGCAGCATCATCAC- 3'<br>R: 5' -ACGATGGTGATCTTGGCCTTGG- 3'
β-actin	F: 5' -CCTTCTTGGGTATGGAATCCTGTG- 3'<br>R: 5' -CAGCACTGTGTTGGCATAGAGG- 3'

Supplementary Table S2. The genomic features of *A. muciniphila* 139 and *A. muciniphila* ATCC

Feature	*A. muciniphila* ATCC	*A. muciniphila* 139
Genome size (bp)	2,664,100 bp	2,801,917 bp
G+C content	55.74%	55.80%
Number of tRNA genes	54	56
Number of 5S rRNA genes	3	3
Number of 16S rRNA genes	3	3
Number of 23S rRNA genes	3	3
Number of Coding Sequences	2182	2342
Protein-coding genes with assigned COG	2005 (91.89%)	2063 (88.09%)

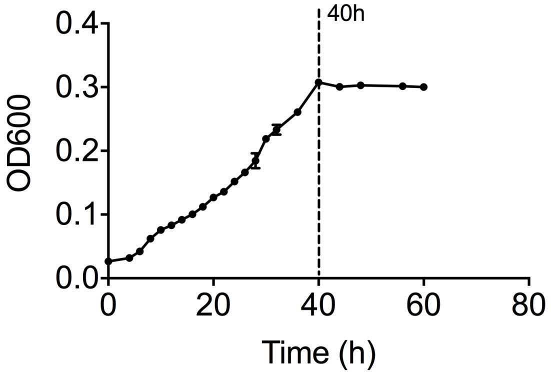

Supplementary Figure S1. The growth curve of *A. muciniphila* 139 in synthetic medium. Data are shown as mean ± s.e.m, n = 3 for each time point. The time for strain 139 growing to stationary phase are also labeled on the plot.

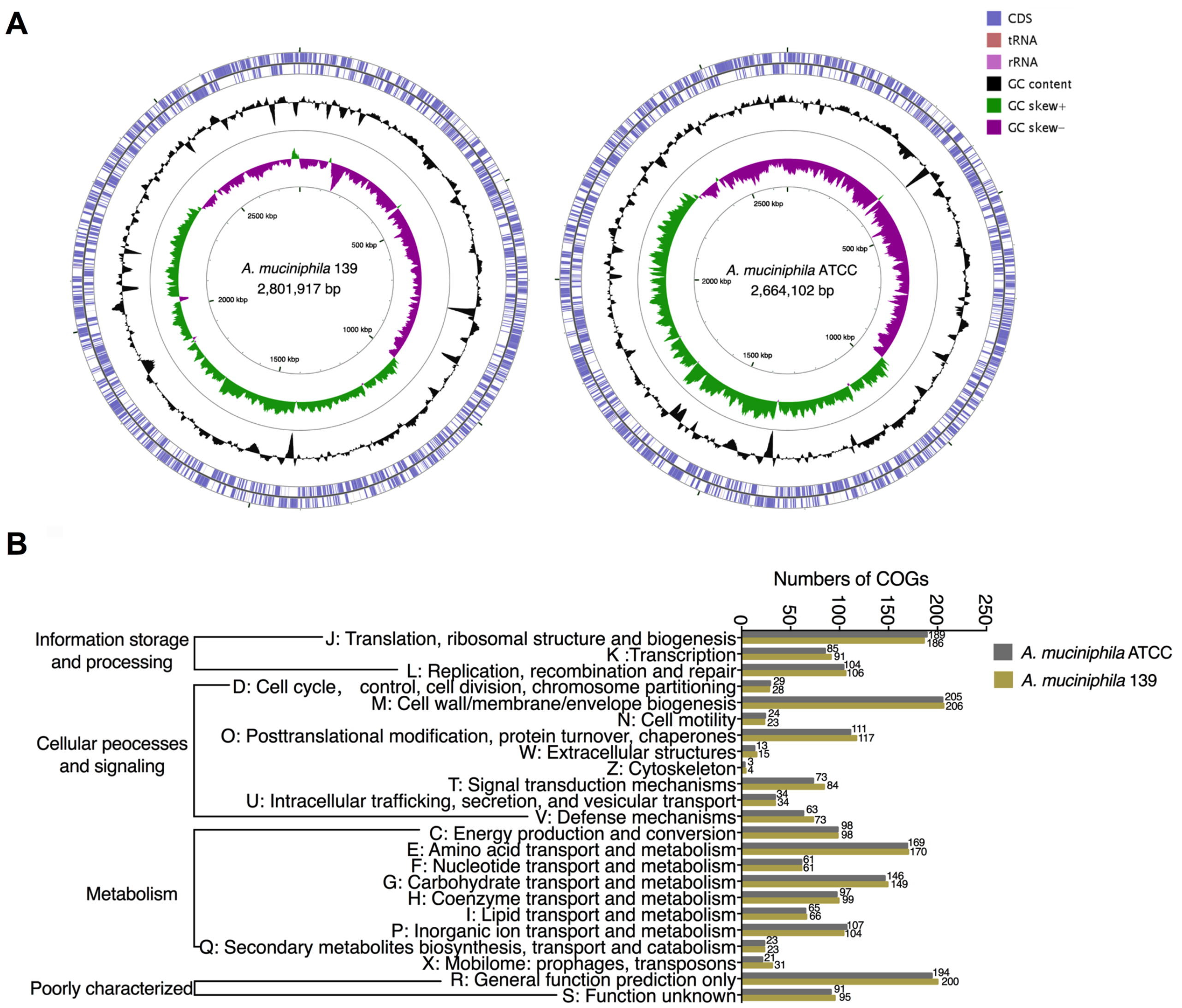

Supplementary Figure S2. Visualization of genomic features of two strains of *A. muciniphila*. **(A)** Genome atlas of the two strains of *A. muciniphila*. From inner to outer: circle 1. GC skew (G − C) / (G + C), circle 2. mean centered GC content, circle 3. tRNAs, rRNAs and CDS (reverse and forward strand). **(B)** Functional categories in the clusters of orthologous groups (COGs) analysis of *A. muciniphila* strain ATCC and 139.

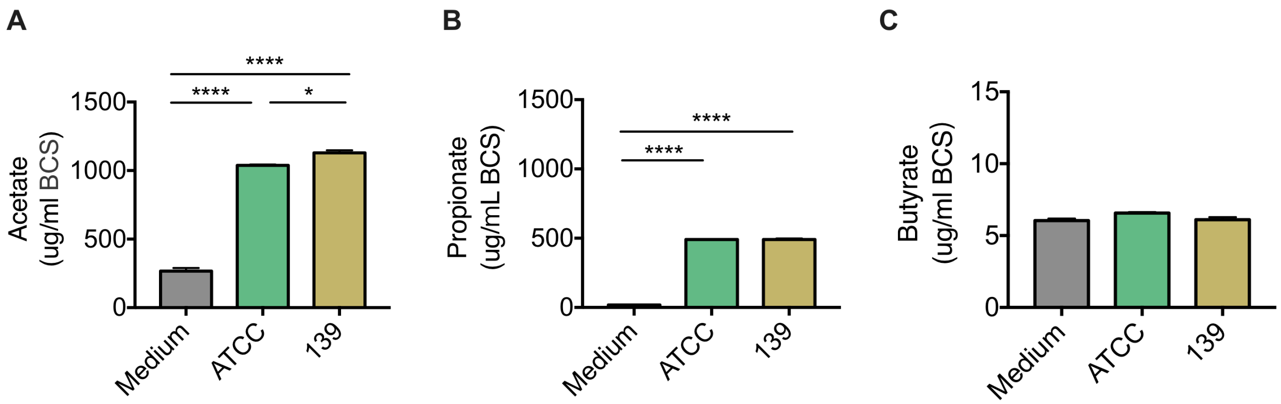

Supplementary Figure S3. Both *A. muciniphila* strains produced acetate and propionate in vitro. The content of acetate **(A)**, propionate **(B)** and butyrate **(C)** in 200μl BCS of two strains. Data are shown as mean ± s.e.m. n = 3 for each group. Values of each group were calculated by one-way analysis of variance (ANOVA) followed by Tukey's post hoc test. * $P < 0.05$, **** $P < 0.0001$.

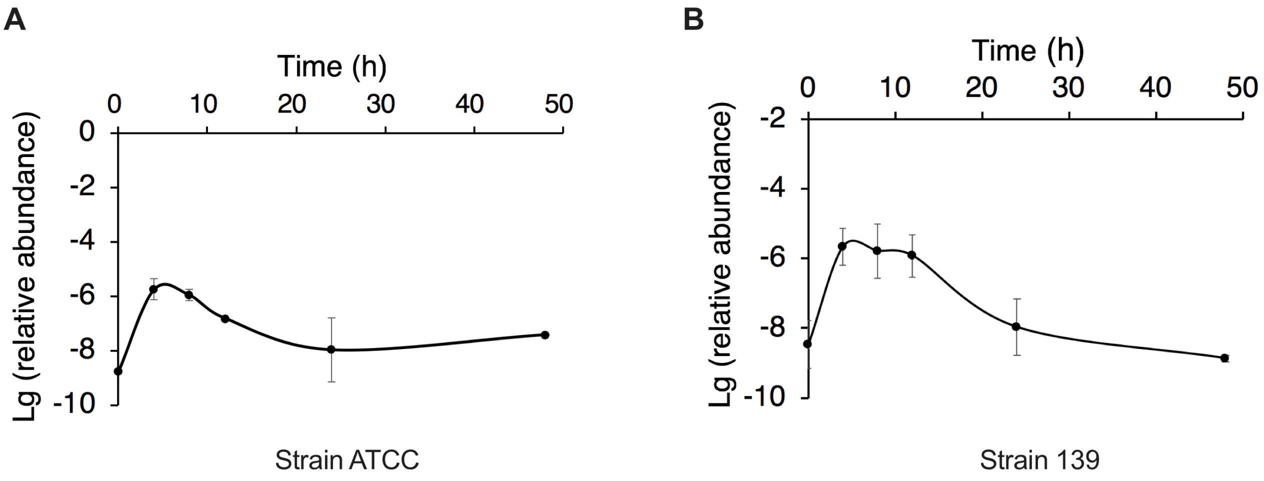

Supplementary Figure S4. Change of relative abundance of fecal *A. muciniphila* after gavaging two strains. (A) Lg (relative abundance) of *A. muciniphila* after gavaging strain ATCC, **(B)** Lg (relative abundance) of *A. muciniphila* after gavaging strain 139. Relative abundance means (copies of *A. muciniphila*)/ (copies of total bacteria). All the data are shown as mean ± s.e.m. n = 3 for each group.

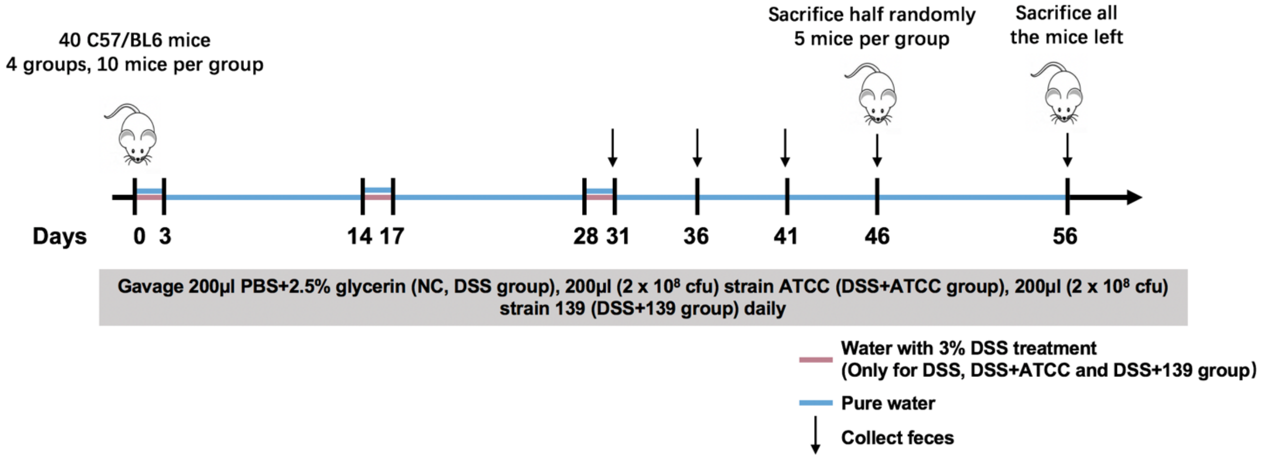

Supplementary Figure S5. Animal experiment design. 40 SPF mice were divided into 4 groups averagely, three groups of mice were treated with 3% DSS to induce chronic colitis, the other one group was healthy control group (NC).

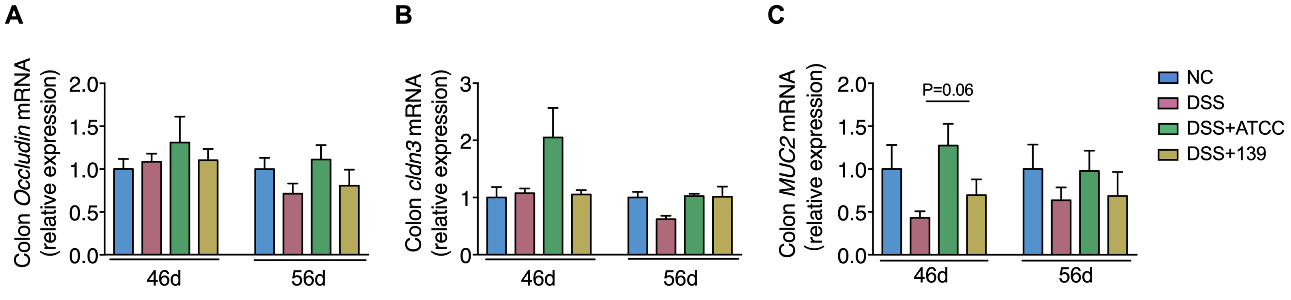

Supplementary Figure S6. *A. muciniphila* ATCC showed tendency to strength the gut permeability of mice. mRNA expression of **(A)** *Occludin*, **(B)** *cldn3* and **(C)** *MUC2*. All the data are shown as mean ± s.e.m. n = 5 for each group. Values of each group were calculated by one-way analysis of variance (ANOVA) followed by Tukey's post hoc test.

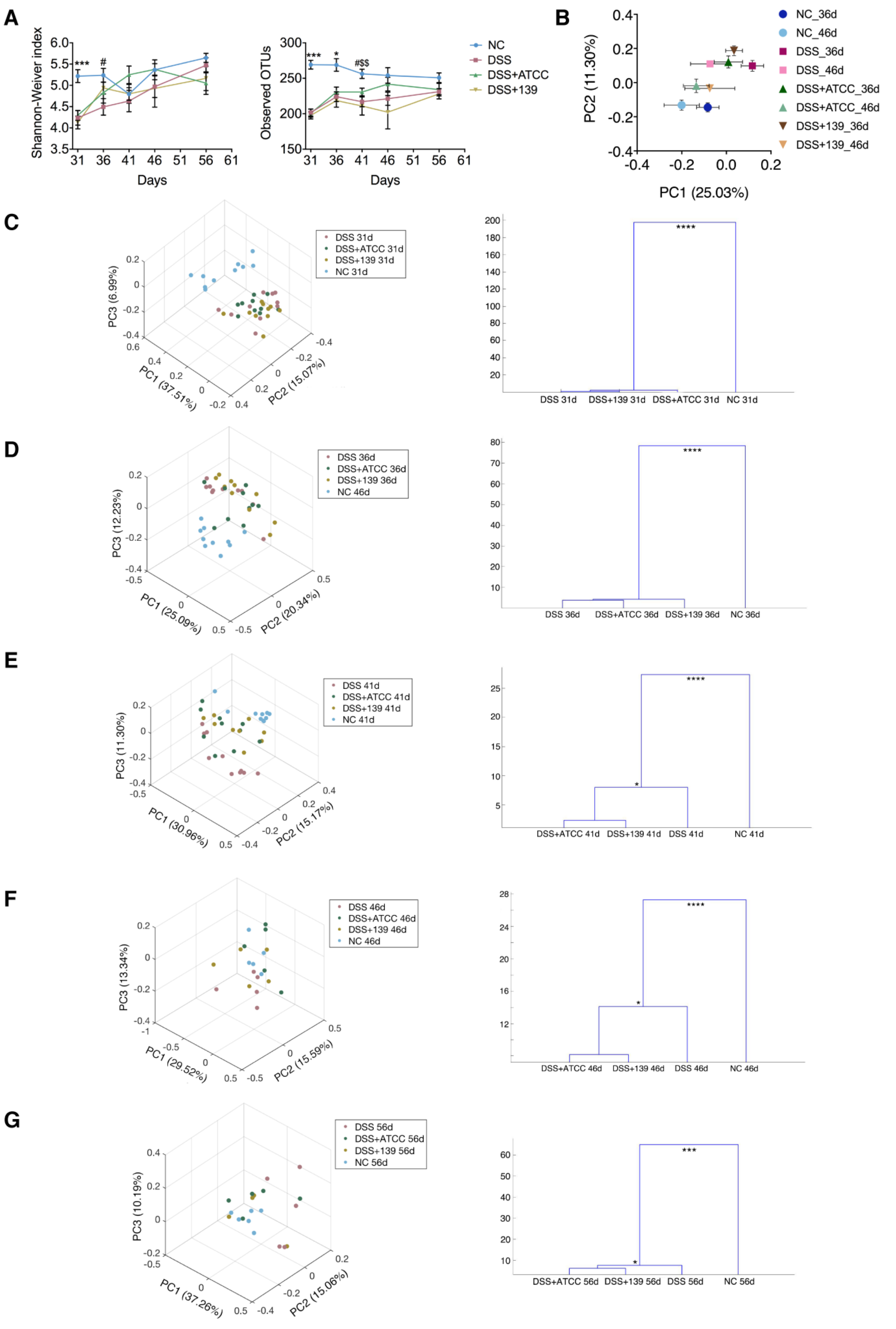
A
Shannon-Weiver index
Days
Observed OTUs
Days
NC
DSS
DSS+ATCC
DSS+139
B
PC2 (11.30%)
PC1 (25.03%)
NC_36d
NC_46d
DSS_36d
DSS_46d
DSS+ATCC_36d
DSS+ATCC_46d
DSS+139_36d
DSS+139_46d
C
DSS 31d
DSS+ATCC 31d
DSS+139 31d
NC 31d
PC3 (6.99%)
PC1 (37.51%)
PC2 (15.07%)
DSS 31d DSS+139 31d DSS+ATCC 31d NC 31d
D
DSS 36d
DSS+ATCC 36d
DSS+139 36d
NC 46d
PC3 (12.23%)
PC1 (25.09%)
PC2 (20.34%)
DSS 36d DSS+ATCC 36d DSS+139 36d NC 36d
E
DSS 41d
DSS+ATCC 41d
DSS+139 41d
NC 41d
PC3 (11.30%)
PC1 (30.96%)
PC2 (15.17%)
DSS+ATCC 41d DSS+139 41d DSS 41d NC 41d
F
DSS 46d
DSS+ATCC 46d
DSS+139 46d
NC 46d
PC3 (13.34%)
PC1 (29.52%)
PC2 (15.59%)
DSS+ATCC 46d DSS+139 46d DSS 46d NC 46d
G
DSS 56d
DSS+ATCC 56d
DSS+139 56d
NC 56d
PC3 (10.19%)
PC1 (37.26%)
PC2 (15.06%)
DSS+ATCC 56d DSS+139 56d DSS 56d NC 56d

Supplementary Figure S7. Both *A. muciniphila* strains changed the diversity and structure of gut microbiota. (A) Shannon index and observed OTUs in gut microbiota of mice. Data are shown as mean ± s.e.m. Values of each group were calculated by one-way analysis of variance (ANOVA) followed by Tukey's post hoc test. ***P<0.001 compared to all three groups treated with DSS, *P<0.05 compared to all three groups treated with DSS, # P<0.05 compared to DSS group, $$ P<0.01 compared to DSS+139 group. **(B)** Bray-Curtis PCoA plot of Day 36 and 46 based on OTU abundance. **(C-G)** PCoA plot and MANOVA test of gut microbiota of mice based on the Bray-Curtis distance at each time point (Day 31, 36, 41, 46, 56). n=10 for each group before 46d time point, n=5 for each group from 46d time point.

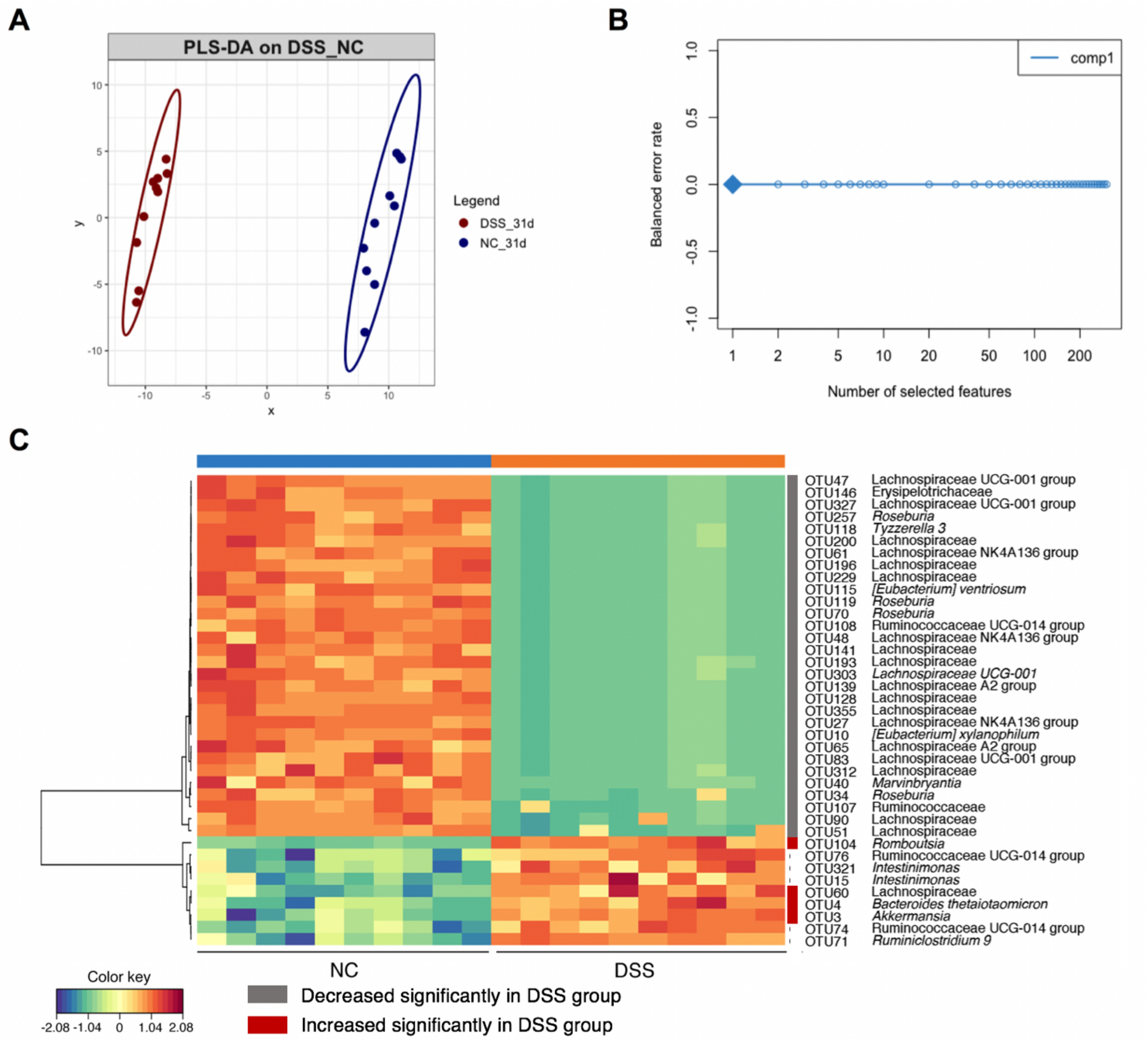

Supplementary Figure S8. 39 OTUs contributed to the form of chronic colitis of mice. sPLS-DA model was used to pick up the OTUs responded to the DSS treatment after the establishment of chronic colitis mice model (Day 31). **(A)** PLS-DA plot of gut microbiota of mice in NC and DSS group. **(B)** Error rate of the sPLS-DA model. **(C)** Heat map of 39 OTUs at Day 31.
